# 2-Oxo-2*H*-chromen-4-yl 4-methyl­benzoate

**DOI:** 10.1107/S1600536813015717

**Published:** 2013-06-12

**Authors:** Akoun Abou, Abdoulaye Djandé, Rita Kakou-Yao, Adama Saba, Abodou Jules Tenon

**Affiliations:** a Laboratoire d’Instrumentation Image et Spectroscopie, DFR–GEE, Institut National Polytechnique Félix Houphouët-Boigny, BP 1093 Yamoussoukro, Côte d’Ivoire; b Laboratoire de Cristallographie et Physique Moléculaire, UFR SSMT, Université Félix Houphouët-Boigny de Cocody, 22 BP 582 Abidjan 22, Côte d’Ivoire; c Laboratoire de Chimie Bio-organique et de Phytochimie, Université de Ouagadougou, 03 BP 7021 Ouagadougou 03, Burkina Faso

## Abstract

The asymmetric unit of the title compound, C_17_H_12_O_4_, consists of two independent mol­ecules. The chromen-2-one ring and the 4-methyl­benzoate side chain are inclined to one another at a dihedral angle of 64.79 (10)° in one mol­ecule and 88.3 (1)° in the other. In the crystal, mol­ecules form *R*_2_^2^(8) centrosymmetric dimers *via* C—H⋯O hydrogen bonds. These dimers are stacked by C—H⋯O hydrogen bonds, resulting in *R*_2_^2^(18) and *R*_3_^2^(16) ring motifs. π–π stacking inter­actions between two parallel chromen-2-one rings, with centroid–centroid distances of 3.743 (1) and 3.771 (1) Å, are also present.

## Related literature

For related structures and background to coumarin derivatives, see: Abou *et al.* (2011 ▶, 2012*a* ▶,*b* ▶). For the biological activity of coumarin derivatives, see: Basanagouda *et al.* (2009 ▶); Vukovic *et al.* (2010 ▶); Emmanuel-Giota *et al.* (2001 ▶). For hydrogen-bond graph-set motifs, see: Bernstein *et al.* (1995 ▶). For π–π stacking inter­actions, see: Janiak (2000 ▶).
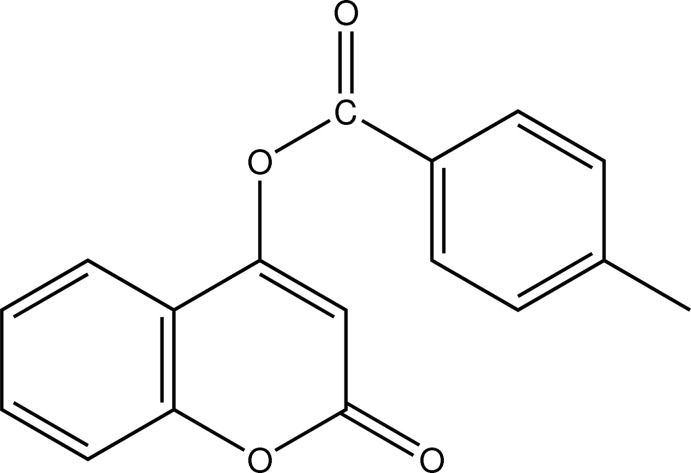


## Experimental

### 

#### Crystal data

C_17_H_12_O_4_
*M* *_r_* = 280.27
Triclinic, 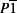

*a* = 9.2790 (5) Å
*b* = 10.7696 (5) Å
*c* = 14.5758 (9) Å
α = 95.274 (2)°
β = 97.875 (2)°
γ = 104.788 (5)°
*V* = 1382.75 (13) Å^3^
*Z* = 4
Mo *K*α radiation
μ = 0.10 mm^−1^
*T* = 298 K
0.35 × 0.20 × 0.20 mm

#### Data collection

Nonius KappaCCD diffractometer
16045 measured reflections
6907 independent reflections
3981 reflections with *I* > 2σ(*I*)
*R*_int_ = 0.055

#### Refinement

*R*[*F* ^2^ > 2σ(*F* ^2^)] = 0.071
*wR*(*F* ^2^) = 0.193
*S* = 1.02
6907 reflections
381 parameters
H-atom parameters constrained
Δρ_max_ = 0.21 e Å^−3^
Δρ_min_ = −0.18 e Å^−3^

### 

Data collection: *COLLECT* (Hooft, 1998 ▶); cell refinement: *DENZO*/*SCALEPACK* (Otwinowski & Minor, 1997 ▶); data reduction: *DENZO*/*SCALEPACK*; program(s) used to solve structure: *SIR2004* (Burla *et al.*, 2005 ▶); program(s) used to refine structure: *SHELXL97* (Sheldrick, 2008 ▶); molecular graphics: *PLATON* (Spek, 2009 ▶); software used to prepare material for publication: *SHELXL97*, *publCIF* (Westrip, 2010 ▶) and *WinGX* (Farrugia, 2012 ▶).

## Supplementary Material

Crystal structure: contains datablock(s) I, global. DOI: 10.1107/S1600536813015717/sj5325sup1.cif

Structure factors: contains datablock(s) I. DOI: 10.1107/S1600536813015717/sj5325Isup2.hkl

Supplementary material file. DOI: 10.1107/S1600536813015717/sj5325Isup3.cml

Additional supplementary materials:  crystallographic information; 3D view; checkCIF report

## Figures and Tables

**Table 1:** Hydrogen-bond geometry (Å, °)

*D*—H⋯*A*	*D*—H	H⋯*A*	*D*⋯*A*	*D*—H⋯*A*
C5*A*—H5*A*⋯O4*B*	0.93	2.55	3.389 (3)	151
C8*A*—H8*A*⋯O2*B* ^i^	0.93	2.52	3.453 (3)	177
C8*B*—H8*B*⋯O2*A* ^i^	0.93	2.50	3.425 (3)	176
C12*A*—H12*A*⋯O2*A* ^i^	0.93	2.59	3.498 (3)	167

Symmetry code: (i) 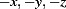.

**Fig. 1.**
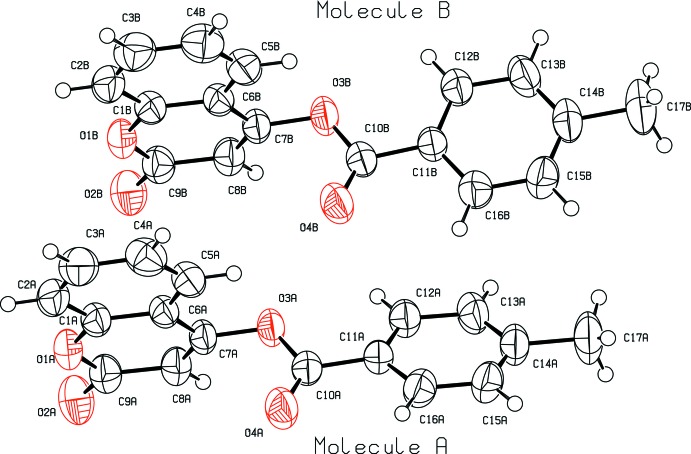
The molecular structure of (I) showing the atomic labeling scheme with displacement ellipsoids drawn at the 50% probability level. H atoms are shown as spheres of arbitary radius.

**Fig. 2.**
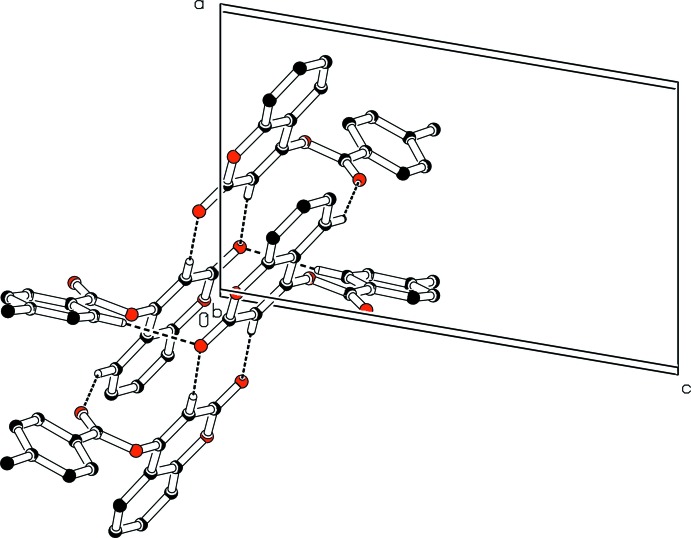
Crystal packing, showing the R22(8) centrosymmetric dimers stacked by hydrogen bonds to form R22(18) and R32(16) ring motifs. Dashed lines indicate hydrogen bonds. H atoms not involved in hydrogen bonds have been omitted for clarity.

**Fig. 3.**
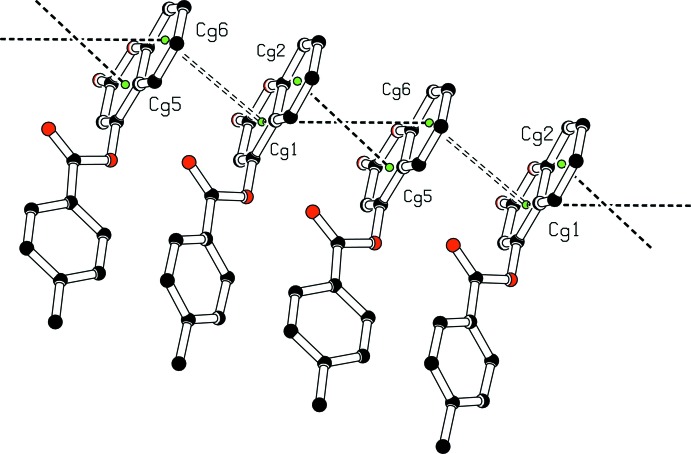
A view of the crystal packing, showing π···π stacking interactions (dashed lines). The green dots are centroids of rings. H atoms have been omitted for clarity.

## supplementary crystallographic information

### Comment

Coumarins and their derivatives constitute one of the major classes of naturally occurring compounds and interest in their chemistry continues unabated because of their usefulness as biologically active agents. They also form the core of several molecules of pharmaceutical importance. Coumarin and its derivatives have been reported to serve as anti-bacterial (Basanagouda *et al.*, 2009), anti-oxidant (Vukovic *et al.*, 2010) and anti-inflammatory agents (Emmanuel-Giota *et al.*, 2001). In view of their importance and as a continuation of our work on the crystal structure analysis of coumarin derivatives (Abou *et al.*, 2011; 2012*a*,*b*), the title ester, (I), C_17_H_12_O_5_ has been synthesized and its molecular and crystal structure is reported herein.

The two independent molecules in the asymmetric unit of the title compound and the atomic labeling scheme are shown in Fig. 1. In these structures, the bond lengths in both independent molecules are comparable to those observed in related structures (Abou *et al.*, 2011; 2012*a*,*b*). Also, the ten-membered chromen-2-one ring systems (O1A/C1A-C9A, O1B/C1B-C9B) of both independent molecules are essentially planar [the maximum deviation from planarity being respectively 0.014 (3) Å for atoms C2A and C4A (molecule A), and -0.010 (2) Å for atom O1B (molecule B)]. In the asymmetric unit, the two chromen-2-one ring systems are parallel displaced, as evidenced by the dihedral angle of 1.25 (7)° between them. In addition, the planar 4-methylbenzoate moieties of the two independent molecules are tilted with respect to one another with a dihedral angle of 26.14 (12)° between them. Furthermore, the angles between the chromen-2-one ring planes and the 4-methylbenzoate side chains of the two independent molecules are inclined at dihedral angles of 64.79 (10)° for molecule A and 88.3 (1)° for molecule B.

In the crystal, we observe the formation of R_2_^2^(8) centrosymmetric dimers (Bernstein *et al.*, 1995) between A and B molecules via C8A—H8A···O2B and C8B—H8B···O2A hydrogen bonds while two adjacent B molecules form similar rings through C2B—H2B···O1B contacts. The dimers are linked to each other by C12A—H12A···O2A^i^ and C12A^i^–H12^i^···O2A hydrogen bonds to form an R_2_^2^(18) ring motif and C5A–H5A···O4B, C8B—H8B···O2A^i^ and C12A—H12A···O2A^i^ contacts [symmetry operation i: - x, - y, - z] result in an R_3_^2^(16) ring motif (Table 1, Fig.2). The supramolecular aggregation is completed by the presence of π···π stacking interactions between two parallel chromen-2-one rings; in the latter, the centroid···centroid distances, Cg1···Cg6 (x - 1, y, z) = 3.771 (1), Cg2···Cg5 = 3.7433 (13) Å, where Cg1, Cg2, Cg5 and Cg6 are centroids of the O1A/C1A/C6A-C9A, C1A/C2A-C6A, O1B/C1B/C6B-C9B and C1B/C2B-C6B rings respectively, are both less than 3.8 Å, the maximum regarded as reasonable for π···π interactions (Janiak, 2000) (Fig. 3).

### Experimental

To a solution of *p*-toluoyl chloride (40 mmole) in dried tetrahydrofuran (150 ml), was added dried triethylamine (120 mmole) and 4-hydroxycoumarin (40 mmole) in small portions over 30 min.The mixture was then refluxed for 3 h and poured into 300 ml of chloroform. The solution was acidified with dilute hydrochloric acid until the pH was 2 - 3. The organic layer was extracted, washed with water, dried over MgSO_4_ and the solvent removed. The crude product was recrystallized from a chloroform-acetone (1/3, *v*/*v*) mixture. Colourless crystals of the title compound were obtained in a good yield: 76%; *M*.pt. 393 K.

### Refinement

H atoms were placed in calculated positions [C—H = 0.93 (aromatic) or 0.96 Å (methyl group)] and refined using a riding model approximation with *U*_iso_(H) constrained to 1.2 (aromatic) or 1.5 (methyl) times *U*_eq_ of the respective parent atom.

### Crystal data

**Table:**

C_17_H_12_O_4_	*Z* = 4
*M**_r_* = 280.27	*F*(000) = 584
Triclinic, *P*1	*D*_x_ = 1.346 Mg m^−^^3^
Hall symbol: -P 1	Melting point: 393 K
*a* = 9.2790 (5) Å	Mo *K*α radiation, λ = 0.71073 Å
*b* = 10.7696 (5) Å	Cell parameters from 16045 reflections
*c* = 14.5758 (9) Å	θ = 2.3–29.0°
α = 95.274 (2)°	µ = 0.10 mm^−^^1^
β = 97.875 (2)°	*T* = 298 K
γ = 104.788 (5)°	Prism, colourless
*V* = 1382.75 (13) Å^3^	0.35 × 0.20 × 0.20 mm

### Data collection

**Table:**

Nonius KappaCCD diffractometer	3981 reflections with *I* > 2σ(*I*)
Radiation source: fine-focus sealed tube	*R*_int_ = 0.055
Graphite monochromator	θ_max_ = 29.0°, θ_min_ = 2.3°
φ and ω scans	*h* = 0→12
16045 measured reflections	*k* = −14→13
6907 independent reflections	*l* = −19→19

### Refinement

**Table:**

Refinement on *F*^2^	Primary atom site location: structure-invariant direct methods
Least-squares matrix: full	Secondary atom site location: difference Fourier map
*R*[*F*^2^ > 2σ(*F*^2^)] = 0.071	Hydrogen site location: inferred from neighbouring sites
*wR*(*F*^2^) = 0.193	H-atom parameters constrained
*S* = 1.02	*w* = 1/[σ^2^(*F*_o_^2^) + (0.0648*P*)^2^ + 0.5338*P*] where *P* = (*F*_o_^2^ + 2*F*_c_^2^)/3
6907 reflections	(Δ/σ)_max_ < 0.001
381 parameters	Δρ_max_ = 0.21 e Å^−^^3^
0 restraints	Δρ_min_ = −0.18 e Å^−^^3^
96 constraints	

### Special details

**Table:**

Geometry. All s.u.'s (except the s.u. in the dihedral angle between two l.s. planes) are estimated using the full covariance matrix. The cell s.u.'s are taken into account individually in the estimation of s.u.'s in distances, angles and torsion angles; correlations between s.u.'s in cell parameters are only used when they are defined by crystal symmetry. An approximate (isotropic) treatment of cell s.u.'s is used for estimating s.u.'s involving l.s. planes.
Refinement. Refinement of *F*^2^ against ALL reflections. The weighted *R*-factor *wR* and goodness of fit *S* are based on *F*^2^, conventional *R*-factors *R* are based on *F*, with *F* set to zero for negative *F*^2^. The threshold expression of *F*^2^ > 2σ(*F*^2^) is used only for calculating *R*-factors(gt) *etc*. and is not relevant to the choice of reflections for refinement. *R*-factors based on *F*^2^ are statistically about twice as large as those based on *F*, and *R*- factors based on ALL data will be even larger.

### Fractional atomic coordinates and isotropic or equivalent isotropic displacement parameters (Å^2^)

**Table:**

	*x*	*y*	*z*	*U*_iso_*/*U*_eq_	
O3A	0.11504 (16)	0.06541 (13)	0.19401 (10)	0.0531 (4)	
O1A	0.01309 (17)	0.34133 (13)	0.03537 (10)	0.0563 (4)	
O3B	0.59037 (18)	0.04620 (14)	0.18431 (10)	0.0586 (4)	
O1B	0.48955 (17)	0.32568 (14)	0.02929 (10)	0.0571 (4)	
C6A	0.1773 (2)	0.28926 (19)	0.16105 (14)	0.0465 (5)	
C7A	0.0784 (2)	0.16012 (18)	0.14343 (14)	0.0470 (5)	
O4A	0.0360 (2)	0.14818 (16)	0.31869 (12)	0.0733 (5)	
O4B	0.4891 (2)	0.11458 (17)	0.30383 (12)	0.0769 (5)	
C8A	−0.0434 (2)	0.1250 (2)	0.07677 (14)	0.0529 (5)	
H8A	−0.1043	0.0402	0.0675	0.063*	
O2B	0.28103 (19)	0.18531 (17)	−0.04626 (11)	0.0718 (5)	
C11B	0.5954 (2)	−0.06380 (19)	0.31683 (14)	0.0475 (5)	
C1A	0.1380 (2)	0.37749 (19)	0.10450 (14)	0.0486 (5)	
O2A	−0.1896 (2)	0.19687 (17)	−0.04247 (11)	0.0747 (5)	
C6B	0.6536 (2)	0.26919 (19)	0.15259 (14)	0.0485 (5)	
C12A	0.1485 (2)	−0.1468 (2)	0.27689 (16)	0.0554 (5)	
H12A	0.1548	−0.1467	0.2138	0.066*	
C12B	0.6854 (2)	−0.1337 (2)	0.28147 (16)	0.0569 (5)	
H12B	0.7191	−0.1180	0.2253	0.068*	
C11A	0.1175 (2)	−0.04418 (19)	0.32675 (14)	0.0474 (5)	
C8B	0.4294 (2)	0.1088 (2)	0.06938 (15)	0.0546 (5)	
H8B	0.3672	0.0245	0.0597	0.065*	
C5B	0.7849 (3)	0.3096 (2)	0.21943 (16)	0.0602 (6)	
H5B	0.8122	0.2515	0.2572	0.072*	
C10A	0.0850 (2)	0.0663 (2)	0.28306 (15)	0.0505 (5)	
C7B	0.5523 (2)	0.14119 (19)	0.13453 (14)	0.0488 (5)	
C10B	0.5508 (2)	0.0404 (2)	0.27117 (15)	0.0519 (5)	
C9A	−0.0812 (3)	0.2178 (2)	0.01896 (15)	0.0550 (5)	
C14B	0.6753 (3)	−0.2549 (2)	0.41256 (17)	0.0625 (6)	
C5A	0.3081 (2)	0.3320 (2)	0.22844 (16)	0.0570 (5)	
H5A	0.3369	0.2749	0.2666	0.068*	
C2B	0.7047 (3)	0.4859 (2)	0.10754 (17)	0.0621 (6)	
H2B	0.6776	0.5449	0.0706	0.075*	
C14A	0.1632 (3)	−0.2525 (2)	0.41558 (18)	0.0642 (6)	
C2A	0.2240 (3)	0.5053 (2)	0.11509 (17)	0.0607 (6)	
H2A	0.1958	0.5634	0.0776	0.073*	
C13A	0.1701 (3)	−0.2502 (2)	0.32218 (18)	0.0647 (6)	
H13A	0.1897	−0.3198	0.2883	0.078*	
C13B	0.7251 (3)	−0.2278 (2)	0.33042 (18)	0.0672 (6)	
H13B	0.7873	−0.2738	0.3069	0.081*	
C1B	0.6160 (2)	0.3591 (2)	0.09711 (15)	0.0504 (5)	
C16A	0.1124 (3)	−0.0446 (2)	0.42087 (16)	0.0614 (6)	
H16A	0.0937	0.0252	0.4550	0.074*	
C16B	0.5426 (3)	−0.0917 (2)	0.39887 (16)	0.0645 (6)	
H16B	0.4793	−0.0467	0.4222	0.077*	
C9B	0.3917 (3)	0.2034 (2)	0.01344 (15)	0.0553 (5)	
C15B	0.5834 (3)	−0.1862 (2)	0.44628 (18)	0.0704 (7)	
H15B	0.5482	−0.2034	0.5018	0.084*	
C15A	0.1347 (3)	−0.1482 (2)	0.46484 (17)	0.0669 (6)	
H15A	0.1305	−0.1475	0.5283	0.080*	
C3A	0.3513 (3)	0.5444 (2)	0.18176 (19)	0.0688 (7)	
H3A	0.4100	0.6299	0.1894	0.083*	
C3B	0.8335 (3)	0.5225 (2)	0.17352 (19)	0.0710 (7)	
H3B	0.8947	0.6071	0.1809	0.085*	
C4A	0.3943 (3)	0.4586 (2)	0.23823 (18)	0.0676 (6)	
H4A	0.4816	0.4868	0.2828	0.081*	
C4B	0.8738 (3)	0.4349 (3)	0.22938 (18)	0.0706 (7)	
H4B	0.9615	0.4613	0.2738	0.085*	
C17A	0.1883 (4)	−0.3653 (3)	0.4641 (2)	0.0970 (10)	
H17A	0.2489	−0.4077	0.4311	0.145*	
H17B	0.0925	−0.4256	0.4650	0.145*	
H17C	0.2394	−0.3341	0.5270	0.145*	
C17B	0.7220 (4)	−0.3568 (3)	0.4659 (2)	0.0941 (10)	
H17D	0.7936	−0.3151	0.5209	0.141*	
H17E	0.7674	−0.4073	0.4270	0.141*	
H17F	0.6346	−0.4124	0.4837	0.141*	

### Atomic displacement parameters (Å^2^)

**Table:**

	*U*^11^	*U*^22^	*U*^33^	*U*^12^	*U*^13^	*U*^23^
O3A	0.0750 (9)	0.0470 (8)	0.0501 (8)	0.0318 (7)	0.0174 (7)	0.0181 (6)
O1A	0.0718 (10)	0.0490 (8)	0.0528 (9)	0.0237 (7)	0.0061 (7)	0.0169 (7)
O3B	0.0833 (10)	0.0533 (9)	0.0554 (9)	0.0381 (8)	0.0204 (8)	0.0221 (7)
O1B	0.0677 (9)	0.0519 (9)	0.0574 (9)	0.0229 (7)	0.0080 (7)	0.0205 (7)
C6A	0.0551 (11)	0.0456 (11)	0.0471 (11)	0.0241 (9)	0.0141 (9)	0.0115 (9)
C7A	0.0635 (12)	0.0413 (10)	0.0463 (11)	0.0256 (9)	0.0162 (9)	0.0140 (9)
O4A	0.1083 (13)	0.0706 (11)	0.0678 (11)	0.0547 (10)	0.0364 (10)	0.0261 (9)
O4B	0.1064 (14)	0.0820 (12)	0.0737 (11)	0.0626 (11)	0.0372 (10)	0.0318 (9)
C8A	0.0674 (13)	0.0441 (11)	0.0502 (12)	0.0188 (10)	0.0094 (10)	0.0115 (9)
O2B	0.0790 (11)	0.0765 (11)	0.0596 (10)	0.0251 (9)	−0.0017 (8)	0.0161 (8)
C11B	0.0487 (11)	0.0442 (11)	0.0519 (12)	0.0155 (8)	0.0060 (9)	0.0138 (9)
C1A	0.0587 (12)	0.0449 (11)	0.0498 (12)	0.0225 (9)	0.0149 (9)	0.0119 (9)
O2A	0.0893 (12)	0.0706 (11)	0.0604 (10)	0.0251 (9)	−0.0104 (9)	0.0129 (8)
C6B	0.0564 (12)	0.0506 (11)	0.0477 (12)	0.0250 (9)	0.0162 (9)	0.0125 (9)
C12A	0.0650 (13)	0.0490 (12)	0.0562 (13)	0.0203 (10)	0.0103 (10)	0.0140 (10)
C12B	0.0660 (13)	0.0522 (12)	0.0602 (14)	0.0251 (10)	0.0145 (10)	0.0158 (10)
C11A	0.0491 (11)	0.0452 (11)	0.0508 (12)	0.0149 (8)	0.0086 (9)	0.0148 (9)
C8B	0.0677 (13)	0.0468 (11)	0.0533 (13)	0.0199 (10)	0.0119 (10)	0.0123 (10)
C5B	0.0610 (13)	0.0673 (15)	0.0591 (14)	0.0270 (11)	0.0110 (10)	0.0140 (11)
C10A	0.0589 (12)	0.0484 (11)	0.0517 (12)	0.0217 (9)	0.0149 (9)	0.0152 (9)
C7B	0.0637 (13)	0.0470 (11)	0.0472 (11)	0.0276 (9)	0.0174 (9)	0.0162 (9)
C10B	0.0575 (12)	0.0504 (12)	0.0546 (13)	0.0233 (10)	0.0106 (9)	0.0143 (10)
C9A	0.0690 (14)	0.0524 (12)	0.0471 (12)	0.0231 (10)	0.0072 (10)	0.0090 (10)
C14B	0.0656 (14)	0.0467 (12)	0.0716 (16)	0.0135 (10)	−0.0054 (11)	0.0204 (11)
C5A	0.0583 (13)	0.0593 (13)	0.0599 (14)	0.0260 (10)	0.0098 (10)	0.0126 (11)
C2B	0.0731 (15)	0.0505 (13)	0.0728 (16)	0.0234 (11)	0.0255 (12)	0.0200 (11)
C14A	0.0637 (14)	0.0551 (13)	0.0766 (17)	0.0160 (10)	0.0061 (11)	0.0320 (12)
C2A	0.0721 (15)	0.0469 (12)	0.0719 (15)	0.0225 (11)	0.0234 (12)	0.0182 (11)
C13A	0.0791 (16)	0.0478 (12)	0.0753 (17)	0.0274 (11)	0.0139 (12)	0.0184 (11)
C13B	0.0781 (16)	0.0569 (14)	0.0772 (17)	0.0353 (12)	0.0106 (13)	0.0181 (12)
C1B	0.0556 (12)	0.0502 (12)	0.0532 (12)	0.0227 (9)	0.0155 (9)	0.0131 (9)
C16A	0.0754 (15)	0.0605 (14)	0.0558 (14)	0.0235 (11)	0.0196 (11)	0.0188 (11)
C16B	0.0726 (15)	0.0713 (15)	0.0614 (15)	0.0323 (12)	0.0185 (11)	0.0223 (12)
C9B	0.0672 (14)	0.0572 (13)	0.0475 (12)	0.0255 (11)	0.0108 (10)	0.0114 (10)
C15B	0.0786 (16)	0.0749 (16)	0.0636 (15)	0.0232 (13)	0.0127 (12)	0.0314 (13)
C15A	0.0760 (16)	0.0719 (16)	0.0582 (14)	0.0212 (12)	0.0132 (11)	0.0303 (12)
C3A	0.0692 (15)	0.0485 (13)	0.0898 (19)	0.0136 (11)	0.0226 (13)	0.0077 (12)
C3B	0.0689 (15)	0.0584 (14)	0.0841 (18)	0.0114 (12)	0.0204 (13)	0.0054 (13)
C4A	0.0573 (14)	0.0634 (15)	0.0784 (17)	0.0145 (11)	0.0062 (11)	0.0032 (12)
C4B	0.0609 (14)	0.0757 (17)	0.0711 (17)	0.0152 (12)	0.0066 (12)	0.0042 (13)
C17A	0.110 (2)	0.0798 (19)	0.114 (2)	0.0353 (17)	0.0117 (18)	0.0591 (18)
C17B	0.113 (2)	0.0727 (18)	0.100 (2)	0.0330 (16)	−0.0055 (18)	0.0416 (16)

### Geometric parameters (Å, º)

**Table:**

O3A—C10A	1.364 (2)	C5B—C4B	1.374 (3)
O3A—C7A	1.395 (2)	C5B—H5B	0.9300
O1A—C9A	1.372 (3)	C14B—C13B	1.371 (3)
O1A—C1A	1.374 (3)	C14B—C15B	1.374 (3)
O3B—C10B	1.368 (2)	C14B—C17B	1.518 (3)
O3B—C7B	1.396 (2)	C5A—C4A	1.377 (3)
O1B—C1B	1.373 (3)	C5A—H5A	0.9300
O1B—C9B	1.374 (3)	C2B—C3B	1.373 (3)
C6A—C1A	1.397 (3)	C2B—C1B	1.384 (3)
C6A—C5A	1.398 (3)	C2B—H2B	0.9300
C6A—C7A	1.435 (3)	C14A—C13A	1.374 (3)
C7A—C8A	1.333 (3)	C14A—C15A	1.379 (3)
O4A—C10A	1.199 (2)	C14A—C17A	1.512 (3)
O4B—C10B	1.196 (2)	C2A—C3A	1.369 (3)
C8A—C9A	1.444 (3)	C2A—H2A	0.9300
C8A—H8A	0.9300	C13A—H13A	0.9300
O2B—C9B	1.213 (3)	C13B—H13B	0.9300
C11B—C12B	1.377 (3)	C16A—C15A	1.382 (3)
C11B—C16B	1.383 (3)	C16A—H16A	0.9300
C11B—C10B	1.474 (3)	C16B—C15B	1.382 (3)
C1A—C2A	1.387 (3)	C16B—H16B	0.9300
O2A—C9A	1.210 (3)	C15B—H15B	0.9300
C6B—C5B	1.396 (3)	C15A—H15A	0.9300
C6B—C1B	1.398 (3)	C3A—C4A	1.389 (3)
C6B—C7B	1.434 (3)	C3A—H3A	0.9300
C12A—C11A	1.379 (3)	C3B—C4B	1.389 (4)
C12A—C13A	1.389 (3)	C3B—H3B	0.9300
C12A—H12A	0.9300	C4A—H4A	0.9300
C12B—C13B	1.387 (3)	C4B—H4B	0.9300
C12B—H12B	0.9300	C17A—H17A	0.9600
C11A—C16A	1.380 (3)	C17A—H17B	0.9600
C11A—C10A	1.478 (3)	C17A—H17C	0.9600
C8B—C7B	1.328 (3)	C17B—H17D	0.9600
C8B—C9B	1.443 (3)	C17B—H17E	0.9600
C8B—H8B	0.9300	C17B—H17F	0.9600
			
C10A—O3A—C7A	117.12 (15)	C3B—C2B—H2B	120.7
C9A—O1A—C1A	122.10 (16)	C1B—C2B—H2B	120.7
C10B—O3B—C7B	116.80 (15)	C13A—C14A—C15A	118.3 (2)
C1B—O1B—C9B	122.14 (16)	C13A—C14A—C17A	121.2 (2)
C1A—C6A—C5A	118.44 (19)	C15A—C14A—C17A	120.4 (2)
C1A—C6A—C7A	116.09 (19)	C3A—C2A—C1A	118.7 (2)
C5A—C6A—C7A	125.46 (19)	C3A—C2A—H2A	120.6
C8A—C7A—O3A	118.30 (18)	C1A—C2A—H2A	120.6
C8A—C7A—C6A	122.48 (18)	C14A—C13A—C12A	121.8 (2)
O3A—C7A—C6A	119.12 (18)	C14A—C13A—H13A	119.1
C7A—C8A—C9A	120.4 (2)	C12A—C13A—H13A	119.1
C7A—C8A—H8A	119.8	C14B—C13B—C12B	121.8 (2)
C9A—C8A—H8A	119.8	C14B—C13B—H13B	119.1
C12B—C11B—C16B	119.31 (19)	C12B—C13B—H13B	119.1
C12B—C11B—C10B	123.22 (19)	O1B—C1B—C2B	116.85 (19)
C16B—C11B—C10B	117.47 (19)	O1B—C1B—C6B	121.46 (19)
O1A—C1A—C2A	116.91 (19)	C2B—C1B—C6B	121.7 (2)
O1A—C1A—C6A	121.55 (18)	C11A—C16A—C15A	120.5 (2)
C2A—C1A—C6A	121.5 (2)	C11A—C16A—H16A	119.8
C5B—C6B—C1B	118.4 (2)	C15A—C16A—H16A	119.8
C5B—C6B—C7B	125.57 (19)	C15B—C16B—C11B	120.3 (2)
C1B—C6B—C7B	116.06 (19)	C15B—C16B—H16B	119.9
C11A—C12A—C13A	119.2 (2)	C11B—C16B—H16B	119.9
C11A—C12A—H12A	120.4	O2B—C9B—O1B	116.8 (2)
C13A—C12A—H12A	120.4	O2B—C9B—C8B	126.1 (2)
C11B—C12B—C13B	119.3 (2)	O1B—C9B—C8B	117.19 (19)
C11B—C12B—H12B	120.3	C14B—C15B—C16B	120.9 (2)
C13B—C12B—H12B	120.3	C14B—C15B—H15B	119.5
C12A—C11A—C16A	119.53 (19)	C16B—C15B—H15B	119.5
C12A—C11A—C10A	122.71 (19)	C14A—C15A—C16A	120.6 (2)
C16A—C11A—C10A	117.74 (19)	C14A—C15A—H15A	119.7
C7B—C8B—C9B	120.5 (2)	C16A—C15A—H15A	119.7
C7B—C8B—H8B	119.7	C2A—C3A—C4A	121.1 (2)
C9B—C8B—H8B	119.7	C2A—C3A—H3A	119.5
C4B—C5B—C6B	120.1 (2)	C4A—C3A—H3A	119.5
C4B—C5B—H5B	120.0	C2B—C3B—C4B	120.9 (2)
C6B—C5B—H5B	120.0	C2B—C3B—H3B	119.6
O4A—C10A—O3A	122.06 (18)	C4B—C3B—H3B	119.5
O4A—C10A—C11A	125.86 (19)	C5A—C4A—C3A	120.2 (2)
O3A—C10A—C11A	112.07 (17)	C5A—C4A—H4A	119.9
C8B—C7B—O3B	119.35 (19)	C3A—C4A—H4A	119.9
C8B—C7B—C6B	122.58 (19)	C5B—C4B—C3B	120.4 (2)
O3B—C7B—C6B	117.97 (18)	C5B—C4B—H4B	119.8
O4B—C10B—O3B	121.76 (18)	C3B—C4B—H4B	119.8
O4B—C10B—C11B	126.1 (2)	C14A—C17A—H17A	109.5
O3B—C10B—C11B	112.09 (17)	C14A—C17A—H17B	109.5
O2A—C9A—O1A	116.75 (19)	H17A—C17A—H17B	109.5
O2A—C9A—C8A	125.9 (2)	C14A—C17A—H17C	109.5
O1A—C9A—C8A	117.37 (19)	H17A—C17A—H17C	109.5
C13B—C14B—C15B	118.3 (2)	H17B—C17A—H17C	109.5
C13B—C14B—C17B	121.1 (2)	C14B—C17B—H17D	109.5
C15B—C14B—C17B	120.6 (2)	C14B—C17B—H17E	109.5
C4A—C5A—C6A	120.0 (2)	H17D—C17B—H17E	109.5
C4A—C5A—H5A	120.0	C14B—C17B—H17F	109.5
C6A—C5A—H5A	120.0	H17D—C17B—H17F	109.5
C3B—C2B—C1B	118.6 (2)	H17E—C17B—H17F	109.5
			
C10A—O3A—C7A—C8A	−105.1 (2)	C1A—O1A—C9A—C8A	−0.8 (3)
C10A—O3A—C7A—C6A	78.5 (2)	C7A—C8A—C9A—O2A	−179.0 (2)
C1A—C6A—C7A—C8A	0.5 (3)	C7A—C8A—C9A—O1A	0.3 (3)
C5A—C6A—C7A—C8A	−178.7 (2)	C1A—C6A—C5A—C4A	0.4 (3)
C1A—C6A—C7A—O3A	176.72 (16)	C7A—C6A—C5A—C4A	179.6 (2)
C5A—C6A—C7A—O3A	−2.5 (3)	O1A—C1A—C2A—C3A	−178.52 (19)
O3A—C7A—C8A—C9A	−176.50 (17)	C6A—C1A—C2A—C3A	1.0 (3)
C6A—C7A—C8A—C9A	−0.2 (3)	C15A—C14A—C13A—C12A	−0.3 (4)
C9A—O1A—C1A—C2A	−179.40 (18)	C17A—C14A—C13A—C12A	−179.5 (2)
C9A—O1A—C1A—C6A	1.1 (3)	C11A—C12A—C13A—C14A	−0.8 (3)
C5A—C6A—C1A—O1A	178.39 (18)	C15B—C14B—C13B—C12B	0.1 (4)
C7A—C6A—C1A—O1A	−0.9 (3)	C17B—C14B—C13B—C12B	−179.0 (2)
C5A—C6A—C1A—C2A	−1.1 (3)	C11B—C12B—C13B—C14B	1.1 (4)
C7A—C6A—C1A—C2A	179.61 (18)	C9B—O1B—C1B—C2B	−178.96 (19)
C16B—C11B—C12B—C13B	−2.1 (3)	C9B—O1B—C1B—C6B	1.4 (3)
C10B—C11B—C12B—C13B	178.3 (2)	C3B—C2B—C1B—O1B	−178.88 (19)
C13A—C12A—C11A—C16A	1.6 (3)	C3B—C2B—C1B—C6B	0.8 (3)
C13A—C12A—C11A—C10A	−176.7 (2)	C5B—C6B—C1B—O1B	179.20 (18)
C1B—C6B—C5B—C4B	−0.1 (3)	C7B—C6B—C1B—O1B	−0.3 (3)
C7B—C6B—C5B—C4B	179.3 (2)	C5B—C6B—C1B—C2B	−0.5 (3)
C7A—O3A—C10A—O4A	−3.5 (3)	C7B—C6B—C1B—C2B	−179.91 (19)
C7A—O3A—C10A—C11A	175.57 (17)	C12A—C11A—C16A—C15A	−1.4 (3)
C12A—C11A—C10A—O4A	168.4 (2)	C10A—C11A—C16A—C15A	177.0 (2)
C16A—C11A—C10A—O4A	−9.9 (3)	C12B—C11B—C16B—C15B	1.9 (3)
C12A—C11A—C10A—O3A	−10.7 (3)	C10B—C11B—C16B—C15B	−178.4 (2)
C16A—C11A—C10A—O3A	171.03 (18)	C1B—O1B—C9B—O2B	177.83 (19)
C9B—C8B—C7B—O3B	−176.69 (18)	C1B—O1B—C9B—C8B	−1.9 (3)
C9B—C8B—C7B—C6B	−0.4 (3)	C7B—C8B—C9B—O2B	−178.3 (2)
C10B—O3B—C7B—C8B	−99.2 (2)	C7B—C8B—C9B—O1B	1.5 (3)
C10B—O3B—C7B—C6B	84.4 (2)	C13B—C14B—C15B—C16B	−0.2 (4)
C5B—C6B—C7B—C8B	−179.6 (2)	C17B—C14B—C15B—C16B	178.9 (2)
C1B—C6B—C7B—C8B	−0.2 (3)	C11B—C16B—C15B—C14B	−0.8 (4)
C5B—C6B—C7B—O3B	−3.3 (3)	C13A—C14A—C15A—C16A	0.6 (4)
C1B—C6B—C7B—O3B	176.12 (16)	C17A—C14A—C15A—C16A	179.7 (2)
C7B—O3B—C10B—O4B	−1.4 (3)	C11A—C16A—C15A—C14A	0.3 (4)
C7B—O3B—C10B—C11B	179.56 (17)	C1A—C2A—C3A—C4A	−0.2 (4)
C12B—C11B—C10B—O4B	−170.2 (2)	C1B—C2B—C3B—C4B	−0.6 (4)
C16B—C11B—C10B—O4B	10.1 (3)	C6A—C5A—C4A—C3A	0.4 (3)
C12B—C11B—C10B—O3B	8.8 (3)	C2A—C3A—C4A—C5A	−0.5 (4)
C16B—C11B—C10B—O3B	−170.85 (19)	C6B—C5B—C4B—C3B	0.3 (4)
C1A—O1A—C9A—O2A	178.64 (19)	C2B—C3B—C4B—C5B	0.0 (4)

### Hydrogen-bond geometry (Å, º)

**Table:**

*D*—H···*A*	*D*—H	H···*A*	*D*···*A*	*D*—H···*A*
C5*A*—H5*A*···O4*B*	0.93	2.55	3.389 (3)	151
C8*A*—H8*A*···O2*B*^i^	0.93	2.52	3.453 (3)	177
C8*B*—H8*B*···O2*A*^i^	0.93	2.50	3.425 (3)	176
C12*A*—H12*A*···O2*A*^i^	0.93	2.59	3.498 (3)	167

Symmetry code: (i) −*x*, −*y*, −*z*.
